# Flipping the Script: Pharmacologic Myocardial Perfusion Imaging and Percutaneous Coronary Intervention in a Patient With Dextrocardia and Situs Inversus Totalis

**DOI:** 10.7759/cureus.101848

**Published:** 2026-01-19

**Authors:** Gabriela L Lacourt Sosa, Raul Cota Ley, Yomara Huertas Gomez, Rafael Rivera-Berrios

**Affiliations:** 1 Medicine, Ponce Health Sciences University, Ponce, PRI; 2 Cardiology, Centro Médico Episcopal San Lucas, Ponce, PRI; 3 Internal Medicine Residency Program, Centro Médico Episcopal San Lucas, Ponce, PRI; 4 Interventional Cardiology, Centro Médico Episcopal San Lucas, Ponce, PRI

**Keywords:** dextrocardia, interventional cardiology, primary percutaneous coronary intervention (pci), situs inversus totalis, situs inversus with dextrocardia

## Abstract

Situs inversus totalis (SIT) is an extremely rare congenital abnormality in which the positions of the body’s organs are mirrored. It can be accompanied by dextrocardia, where the heart is located in the right thorax with its apex pointing to the right side of the body. Its rarity poses diagnostic and therapeutic challenges, particularly in patients with coronary artery disease (CAD). We report the case of a 74-year-old woman who presented with dyspnea. Her medical history included hypertension, diabetes mellitus, hypothyroidism, and dextrocardia with SIT, incidentally diagnosed at age 45 following two cerebrovascular accidents.

She underwent pharmacologic myocardial perfusion imaging (MPI) with 99mTc-MIBI and adenosine, which revealed mild-to-moderate left ventricular dilation; a medium-sized, moderate-intensity lateral wall perfusion defect during stress; partial lateral wall redistribution at rest; and a preserved left ventricular ejection fraction of 89%. Subsequent left and right heart catheterization (LHC and RHC) and percutaneous coronary intervention (PCI) were performed via femoral access, with successful stent deployment despite the mirrored coronary anatomy. This case underscores the importance of anatomical awareness, imaging interpretation skills, and procedural adaptability when performing coronary interventions in patients with SIT and dextrocardia.

## Introduction

Situs inversus totalis (SIT) is a rare congenital anomaly where the body’s thoracic and abdominal organs are mirrored in position [1]. Importantly, patients with SIT have a condition called dextrocardia, where the heart is located on the right side of the thorax, with its apex pointing to the right side of the body. With a prevalence of 1/10,000 live births, its extreme rarity creates important diagnostic and therapeutic challenges when managing patients with SIT [2]. 

Patients with SIT have a similar risk of coronary artery disease (CAD) as the general population and may require diagnostic coronary angiography or percutaneous coronary interventions (PCI) when significant coronary lesions are present [3]. However, the mirror-image coronary anatomy complicates both noninvasive and invasive cardiac procedures. Standard imaging orientations, such as electrocardiography leads, echocardiographic views, and nuclear myocardial perfusion imaging (MPI) planes, may need to be adjusted to avoid misinterpretation. Similarly, PCI requires modifications in catheter selection, manipulation, and fluoroscopic angulations to safely and effectively access coronary arteries.

Several studies have described the technical adaptations necessary for performing PCI in patients with dextrocardia. For instance, fluoroscopic projections are often inverted: right anterior oblique (RAO) views substitute for left anterior oblique (LAO) views, and vice versa, while maintaining standard craniocaudal angulations. Additionally, guiding catheters must be rotated in a mirrored direction to engage the coronary ostia correctly, reflecting the reversed orientation of the coronary tree [4]. These adjustments, sometimes referred to as the “double inversion technique,” allow operators to perform PCI safely, achieve adequate stent deployment, and maintain procedural efficiency despite the anatomical challenges.

Understanding the unique considerations in patients with SIT and dextrocardia is essential, not only for interventional cardiologists but also for imaging specialists and clinicians involved in pre-procedural planning. Careful planning, awareness of mirror-image anatomy, and adaptation of standard techniques are critical to minimizing procedural complications and ensuring successful outcomes.

In this report, we present a 74-year-old woman with SIT and dextrocardia who underwent pharmacologic MPI followed by PCI. Her case highlights the clinical and technical considerations necessary for the safe evaluation and management of CAD in patients with this rare congenital anomaly.

## Case presentation

**Figure 1:**
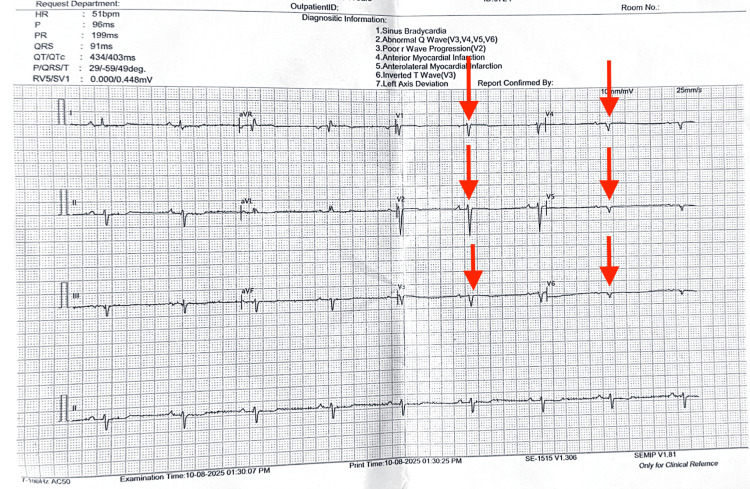
Baseline ECG showing sinus bradycardia with T-wave inversions in anterolateral leads. Red arrows: Poor R-wave progression in precordial leads, consistent with dextrocardia.

A 74-year-old woman presented with progressive dyspnea. Her past medical history was significant for hypertension, diabetes mellitus, hypothyroidism, and dextrocardia with SIT, which had been incidentally diagnosed at age 45 following two cerebrovascular accidents. Baseline electrocardiography demonstrated sinus bradycardia with T-wave inversions in the anterolateral leads (Figure 1). Given her symptoms and cardiovascular risk factors, she underwent pharmacologic MPI using 99mTc-MIBI with adenosine infusion, revealing reversible lateral wall ischemia. 

During adenosine infusion, she remained asymptomatic, with blood pressure increasing from 123/71 mmHg to 131/79 mmHg and heart rate increasing from 47 bpm to 82 bpm, without significant ischemic changes on electrocardiogram. Stress imaging revealed mild-to-moderate left ventricular dilation and a medium-sized, moderate-intensity lateral wall perfusion defect. Rest images demonstrated partial lateral wall redistribution, consistent with reversible ischemia. Gated imaging showed preserved left ventricular contractility with a calculated ejection fraction of 89%.

**Figure 2:**
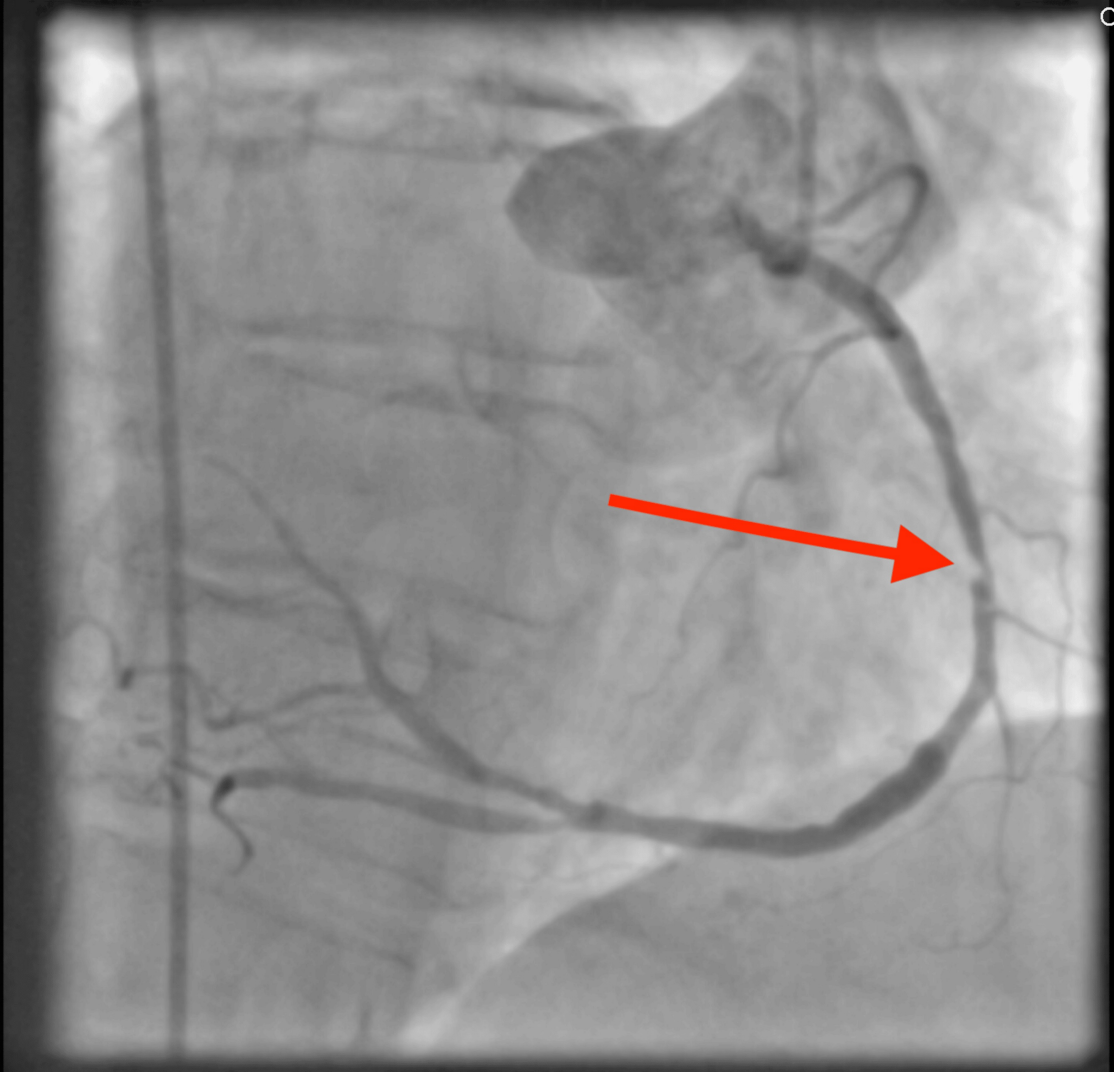
Right coronary angiography demonstrating a 95% obstruction of the right coronary artery. Also notable is the inversion of the aortic root in a PA projection. Red arrow: 95% obstruction.

**Figure 3:**
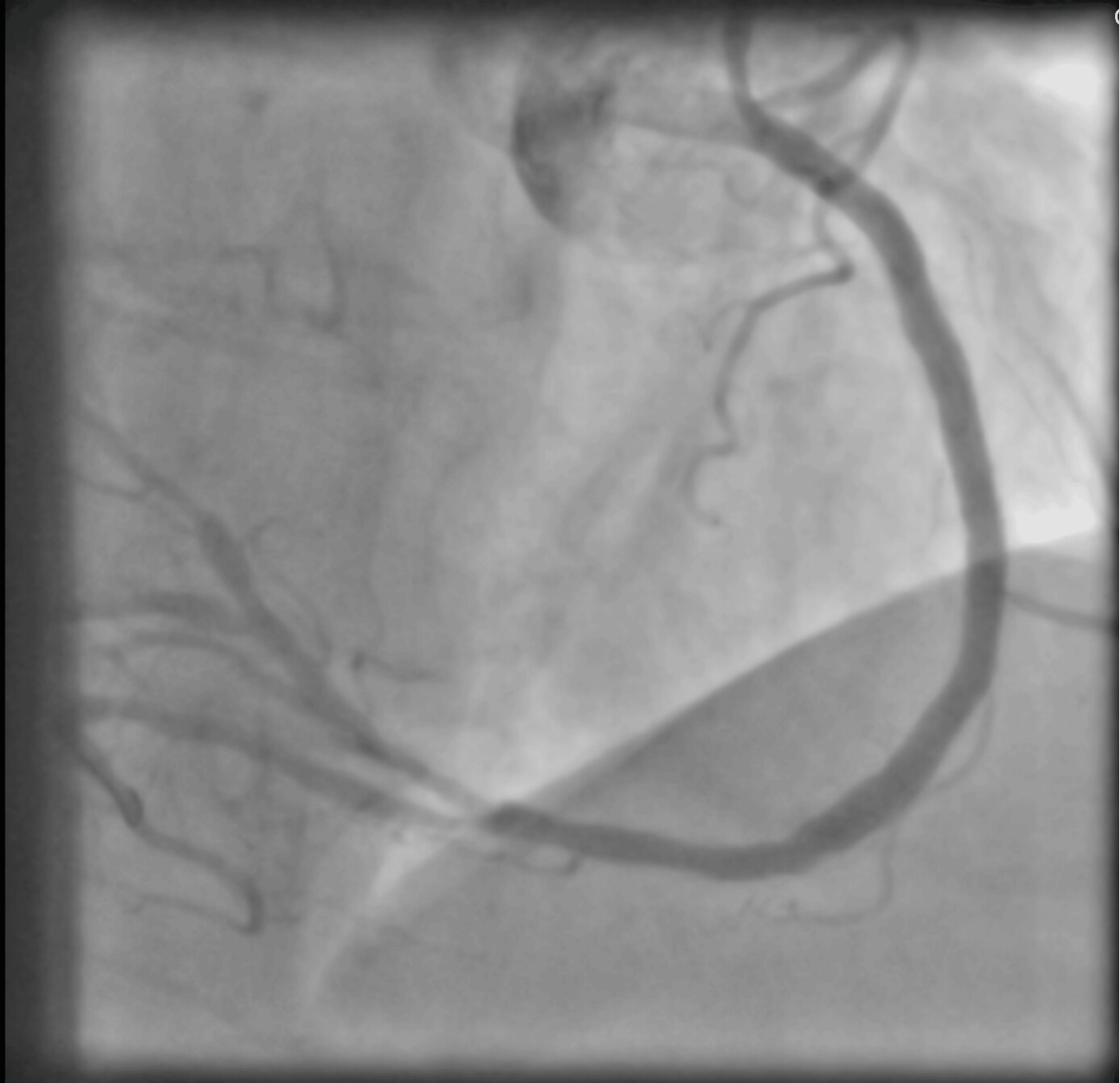
Right coronary artery angiography after stent deployment resulting in 0% residual stenosis and maintained TIMI 3 flow on final angiography.

She subsequently underwent left heart catheterization (LHC) via femoral approach. Following conscious sedation and local anesthesia, a 5-French sheath was placed in the right common femoral artery. A 5-French JL4 diagnostic catheter was advanced over a guidewire to cannulate the left main coronary artery, followed by selective angiography in orthogonal views. The catheter was exchanged for a 5-French JR4 to cannulate the right coronary artery (RCA), and angiography was performed, revealing a 95% mid RCA stenosis (Figure 2). The patient subsequently underwent PCI via femoral access. Due to her mirrored coronary anatomy, fluoroscopic projections were inverted and catheters manipulated in a mirrored fashion to engage the coronary ostia. The RCA lesion was crossed with a 0.014” guidewire, and the vessel was sequentially pretreated with balloon angioplasty, including a 2.5 × 20 mm TREK balloon at 8 atm, a 3.0 × 10 mm WOLVERINE cutting balloon at 6 atm, and a 3.5 × 12 mm NC TREK NEO balloon at 12 atm. Due to the calcified nature of the lesion, rotational atherectomy was performed using a 1.5 mm Rotablator burr to modify the plaque and facilitate stent delivery. Following successful lesion preparation, a 3.5 × 48 mm XIENCE Skypoint drug-eluting stent was deployed in the RCA at 12 atm for six seconds, achieving full lesion coverage. Post-dilation was performed with a 3.5 × 12 mm NC TREK NEO balloon at 12 atm to optimize stent expansion, resulting in 0% residual stenosis and maintained TIMI 3 flow on final angiography (Figure 3).

A 5-French pigtail catheter was then advanced into the left ventricle for ventriculography and hemodynamic assessment, including pull-back pressures across the aortic valve. All catheters were removed, and hemostasis was achieved with manual compression. The patient tolerated the procedure well without complications.

**Figure 4:**
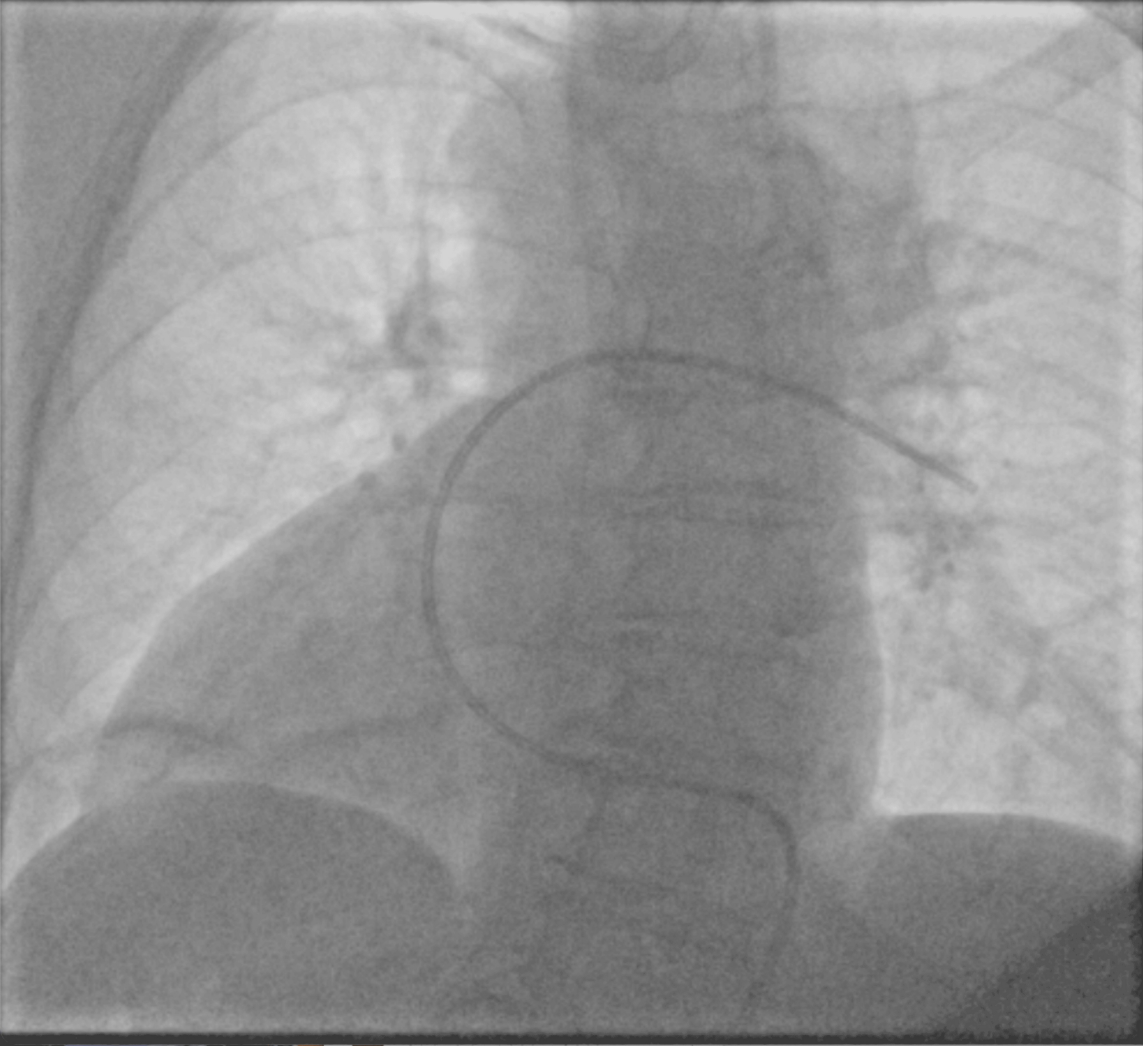
Fluoroscopy from right heart catheterization showing a right-sided and mirrored myocardium due to situs inversus totalis with dextrocardia.

Right heart catheterization (RHC) was also performed via femoral access. A 7-French Swan-Ganz catheter was advanced into the right atrium, measuring a pressure of 17 mmHg, then into the right ventricle (34/18/15 mmHg) and wedge position (17 mmHg) (Figure 4). Pulmonary arterial pressures were 39/28/31 mmHg, cardiac output was 2.52 L/min, cardiac index was 1.62 L/min, and pulmonary vascular resistance was 6 Wood units, demonstrating a combined pre- and post-capillary pulmonary hypertension. The catheter was removed, and hemostasis was achieved without complications.

The patient remained hemodynamically stable and was discharged on guideline-directed medical therapy for CAD, with plans for routine follow-up.

## Discussion

The rarity of SIT with dextrocardia poses significant diagnostic and therapeutic challenges in clinical practice. Because this condition is infrequently encountered, the available literature describing interventional management is limited, leaving physicians with minimal guidance when treating patients with such anatomical variants. Although individuals with SIT have a comparable risk of developing CAD as the general population, the mirror-image orientation of the thoracic and abdominal organs complicates both diagnostic interpretation and interventional procedures such as PCI. Awareness of these variations and the technical adaptations they require is essential to ensure accurate diagnosis and safe procedural outcomes. 

Standard (non-adapted) PCI techniques do not take into account patients with mirrored anatomical variants such as SIT with dextrocardia. The reversal of cardiac and vascular orientation requires interventionalists to modify their standard procedural approach. Physicians must employ alternative strategies, including inverted fluoroscopic imaging during both LHC and RHC, to correctly visualize and engage the coronary arteries. Beyond technical adjustments, the operator’s spatial understanding and procedural orientation must be conceptually reversed to accommodate the patient’s unique anatomy. This cognitive adaptation is as critical as the technical modifications themselves to ensure procedural success and minimize complications.

Other studies have demonstrated that when appropriate procedural modifications are implemented, PCI can be performed safely and effectively in patients with SIT. In a study by Magarkar et al. [5], eight patients with SIT underwent successful femoral PCI without post-procedural complications or significant residual stenosis after operators employed mirrored orientation techniques. These adjustments included reversing the usual catheter manipulation, such as using counterclockwise rotation where a clockwise rotation would normally be applied, and vice versa. Additionally, fluoroscopic imaging was inverted from left to right to accommodate the inverted coronary anatomy. Such findings reinforce that, with proper anatomical understanding and technical adaptation, excellent procedural outcomes are achievable even in complex mirrored variants.

This case underscores the importance of individualized procedural planning and anatomical awareness in patients with congenital variants such as SIT. A clear pre-procedural understanding of the patient’s mirrored coronary anatomy allows for appropriate catheter and projection selection, minimizing procedural time and risk. Collaboration among the cardiology, radiology, and anesthesiology teams is essential to ensure correct image interpretation and safe intervention. Such multidisciplinary coordination is particularly valuable when conventional orientations may lead to misinterpretation or technical difficulty.

Beyond interventional cardiology, diagnostic imaging in SIT requires thoughtful adjustment of conventional protocols. Electrocardiogram lead placement must be reversed to accurately capture cardiac activity, and nuclear imaging planes must be mirrored to prevent false localization of ischemia. In our patient, proper imaging adaptation was crucial to identifying a true lateral wall perfusion defect rather than a technical artifact. This highlights that diagnostic accuracy depends as much on anatomical awareness as on imaging technology itself.

Ultimately, successful management of CAD in patients with SIT and dextrocardia relies on meticulous pre-procedural assessment, technical flexibility, and conceptual reorientation of anatomical understanding. When these elements are applied, PCI can be performed as safely and effectively as in patients with normal cardiac orientation.

## Conclusions

SIT with dextrocardia presents unique diagnostic and interventional challenges due to its mirror-image anatomy. Successful management of CAD in these patients depends on thorough anatomical understanding, appropriate modification of standard techniques, and careful procedural planning. With awareness and adaptation, such as inverted fluoroscopic projections and reversed catheter manipulation, PCI can be performed safely and effectively, achieving outcomes comparable to those in patients with normal anatomy. This case reinforces the importance of clinical vigilance and technical adaptations when encountering rare anatomical variants.
